# Go with the flow and accessorize your drugs

**DOI:** 10.1186/1758-2946-6-S1-O11

**Published:** 2014-03-11

**Authors:** Gisbert Schneider

**Affiliations:** 1 Department of Chemistry and Applied Biosciences, Eidgenössische Technische Hochschule (ETH), Wolfgang-Pauli-Str. 10, 8093 Zürich, Switzerland

## 

The fast pace of drug discovery programs is supported by high-throughput screening campaigns to identify new chemical entities, where the underlying screening compound collections benefit from combinatorial libraries with lead- and drug-like properties. We will discuss recent developments in this field from two perspectives by addressing how to (i) assemble focused combinatorial libraries with desired properties (search problem), and (ii) identify targets for the designed compounds and, vice versa, compounds with desired target activities (scoring problem). Developments from our laboratory will be highlighted, specifically the MAntA (*Molecular Ant Algorithm*) approach for molecular building block prioritization, the target prediction method SPiDER (*SOM-based Prediction of Drug Equivalence Relationships*), and a quantitative Gaussian process model covering several hundred macromolecular targets for compound scoring. Using these algorithms we successfully generated activity-focused combinatorial small molecule and peptide libraries, de-orphaned *de novo* designed molecules, and correctly predicted off-target liabilities of known drugs. Selected examples of these practical applications will be presented, including an integrative application of combinatorial design, microfluidics-assisted synthesis, and activity testing.

